# Supplementary figures and images for: Morphological and transcriptomic evidence for ammonium induction of sexual reproduction in Thalassiosira pseudonana and other centric diatoms
Source: PLoS One. 2017 Jul 7;12(7):e0181098. doi: 10.1371/journal.pone.0181098 (PMC5501676; doi:10.1371/journal.pone.0181098)

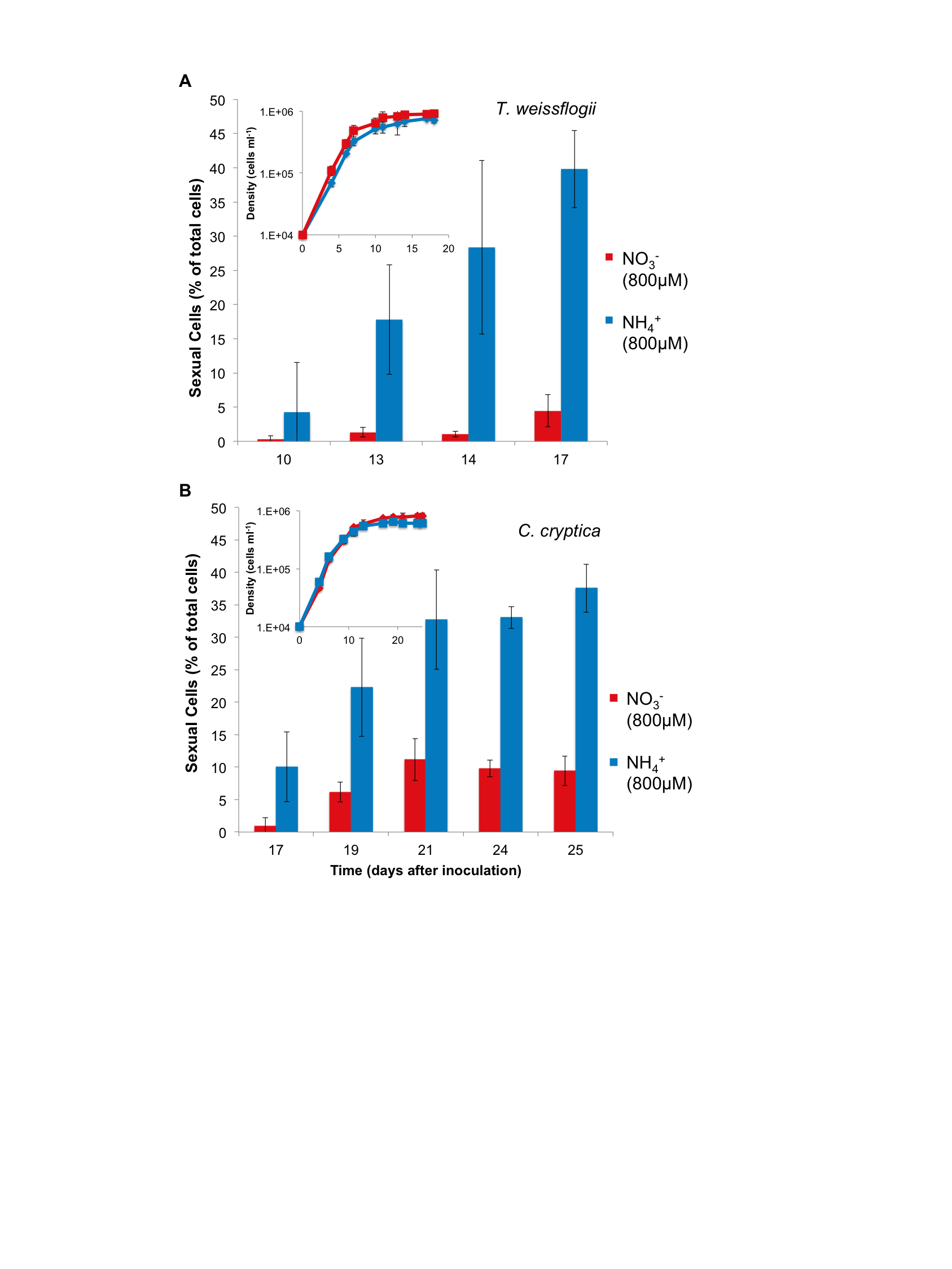

Supplement: S1 Fig — Ammonium induces sexual morphologies in T. weissflogii (A) and C. cryptica (B). Proportion of sexual cells (oogonia and auxospores) relative to the total population in cultures supplemented with NH4Cl or NaNO3. An average of 120 and 107 cells were counted per replicate of T. weissflogii and C. cryptica, respectively, throughout the growth curve, but oogonia and auxospores were only observed beginning in stationary phase; independent cultures n = 3, data are mean values, error bars are s.d.. Inset: corresponding growth curve linking the onset of stationary phase with first appearance of sexual cells on day 10 (A) and 17 (B). (TIF) [file pone.0181098.s001.tif]

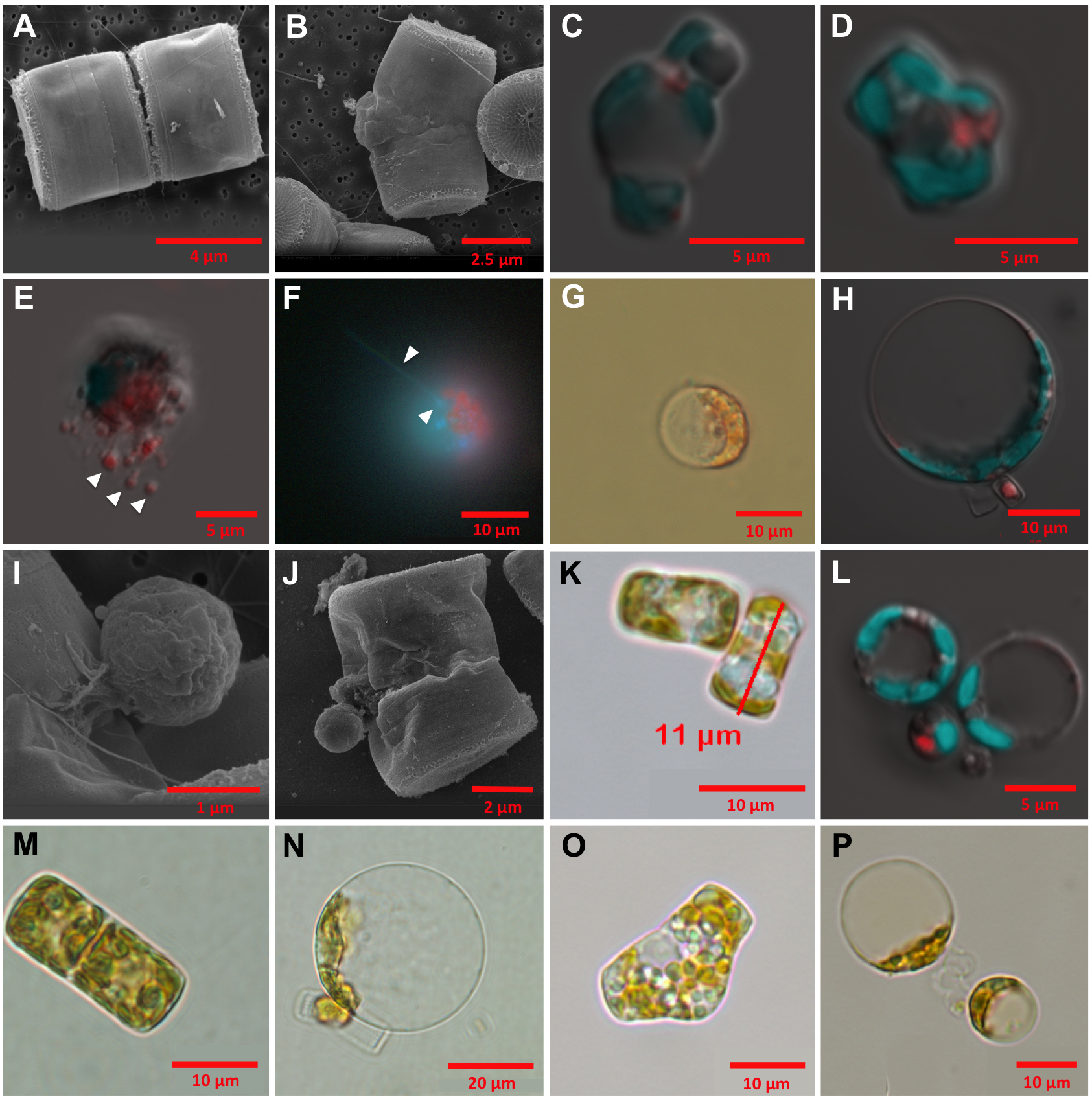

Supplement: S2 Fig — The different life stages in T. pseudonana (A-L), T. weissflogii (M,N) and C. cryptica (O,P). A: SEM of vegetative cells (CCMP1335). B-D: SEM (B) and CFM images of CCMP1335 oogonia, displaying separation of the thecae and expansion of the membrane. E: CFM image of flagellated spermatocytes with stained DNA (arrowheads), F, G. Epifluorescence (F) and LM images of the same view. In F, an active, flagellated spermatocyte (arrowhead) possibly associated with an auxospore surface is revealed by lateral light from fluorescence of DNA (blue) and chlorophyll (red). H,L: Auxospores of CCMP1015 and CCMP1335 respectively (CFM). I,J: Individual spermatocytes attached to oogonia (SEM). K: Initial cells of T. pseudonana CCMP1335 (LM). M,N: T. weissflogii vegetative cells (M; LM) and auxospore (N; LM). O,P: C. cryptica oogonia (O; LM) and auxospores (P; LM). CFM images (C-E, H, L) show fluorescence of DNA in red and chlorophyll in green. (TIF) [file pone.0181098.s002.tif]

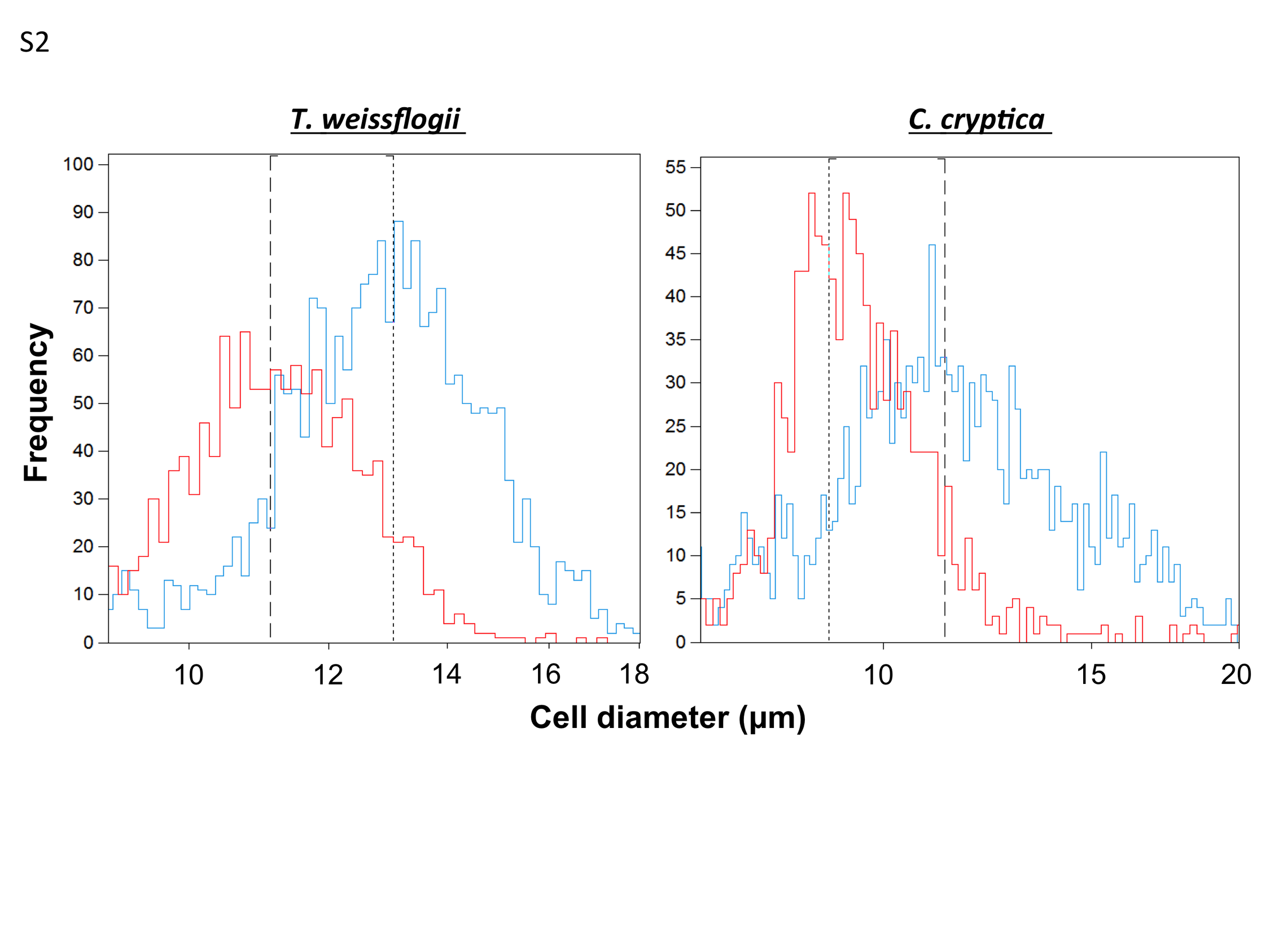

Supplement: S3 Fig — Coulter Counter distributions of cell diameter for T. weissflogii (A) and C. cryptica (B) cultures in exponential phases of growth and maintained in NaNO3 (red) and after two successive 25% transfers to media with ammonium (blue), Each new culture was allowed to remain in stationary phase for three days before the next 25% transfer was made. Single replicates. Dashed lines are the mode for each peak. Cell densities in (A) are 2.2 x 105 ml-1 (NaNO3) and 3.3 x 105 ml-1 (ammonium) and (B) are 1.6 x 106 ml-1 (NaNO3) and 2.2 x 106 ml-1 (ammonium). (TIF) [file pone.0181098.s003.tif]

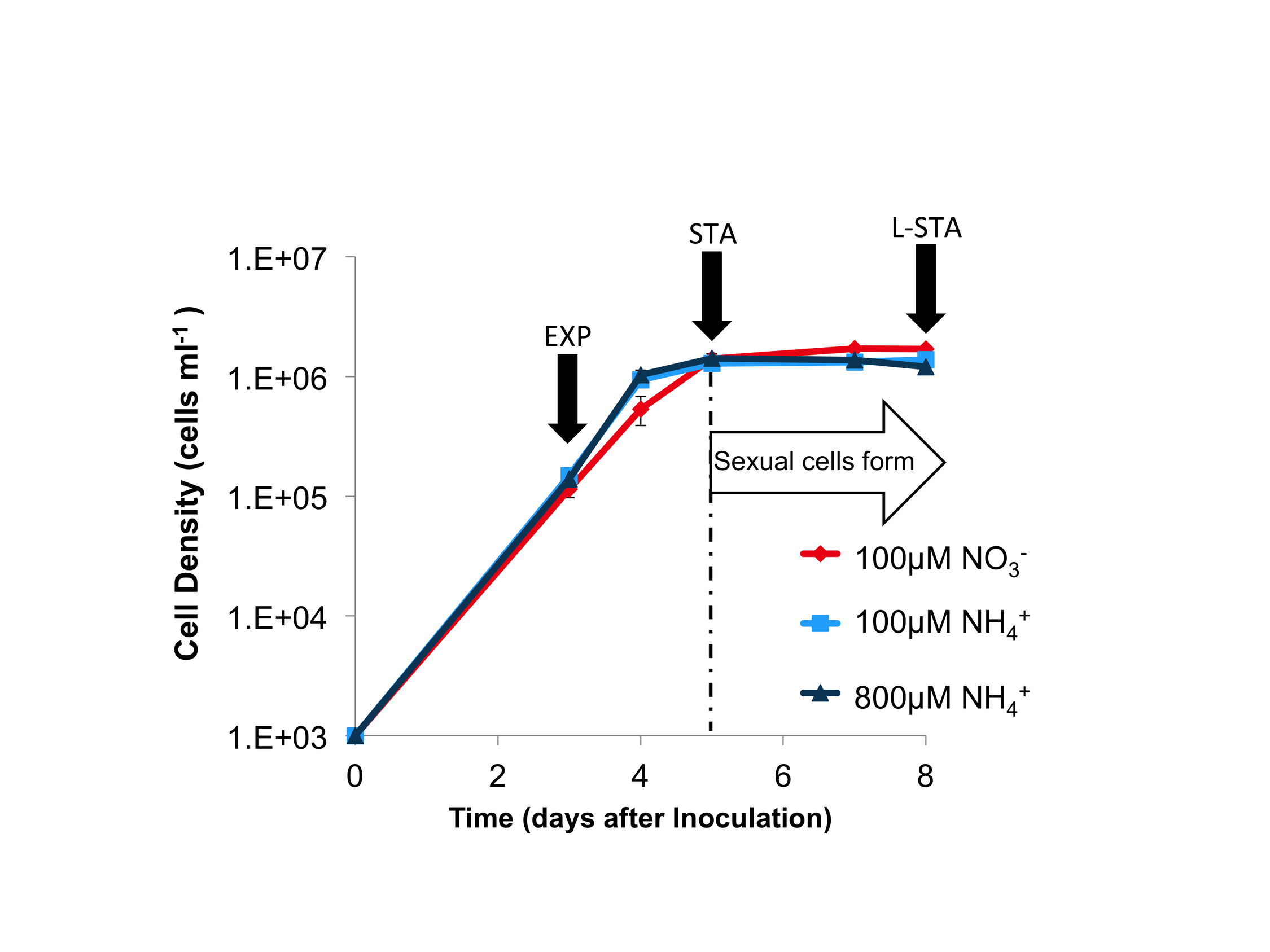

Supplement: S4 Fig — Three independent cultures of each nitrogen treatment were harvested 3, 5, and 8 days after inoculation (down arrows) in exponential (EXP), stationary (STA) and late stationary phases (L-STA). The 100uM NH4+ STA treatment did not yield sufficient RNA for analysis. (TIF) [file pone.0181098.s004.tif]

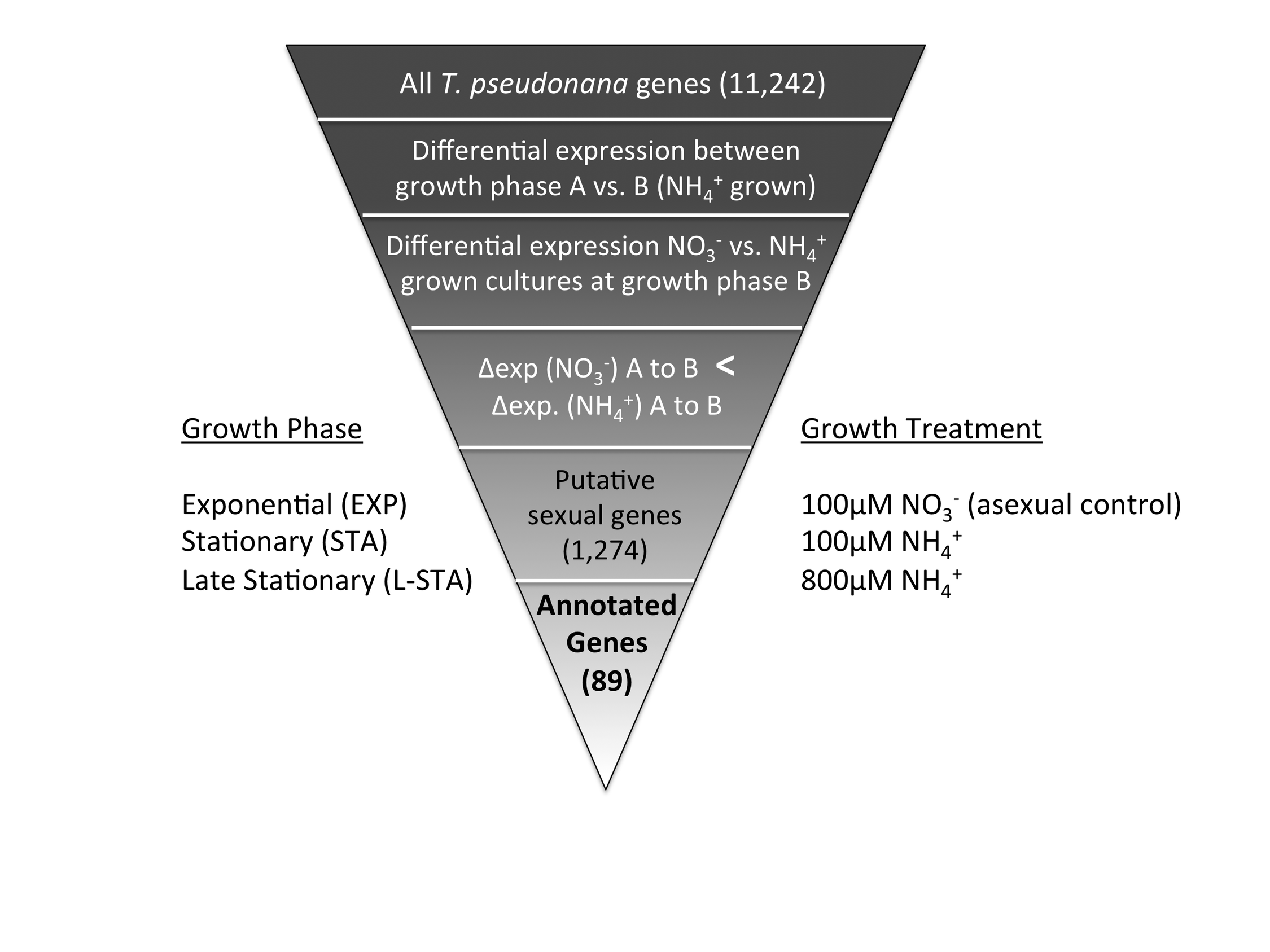

Supplement: S5 Fig — Growth phase A vs. B is EXP vs. STA, EXP vs. L-STA, or STA vs. L-STA, respectively. Δexp is the magnitude of change in gene expression between growth phases for the different nitrogen treatments. (TIF) [file pone.0181098.s005.tif]

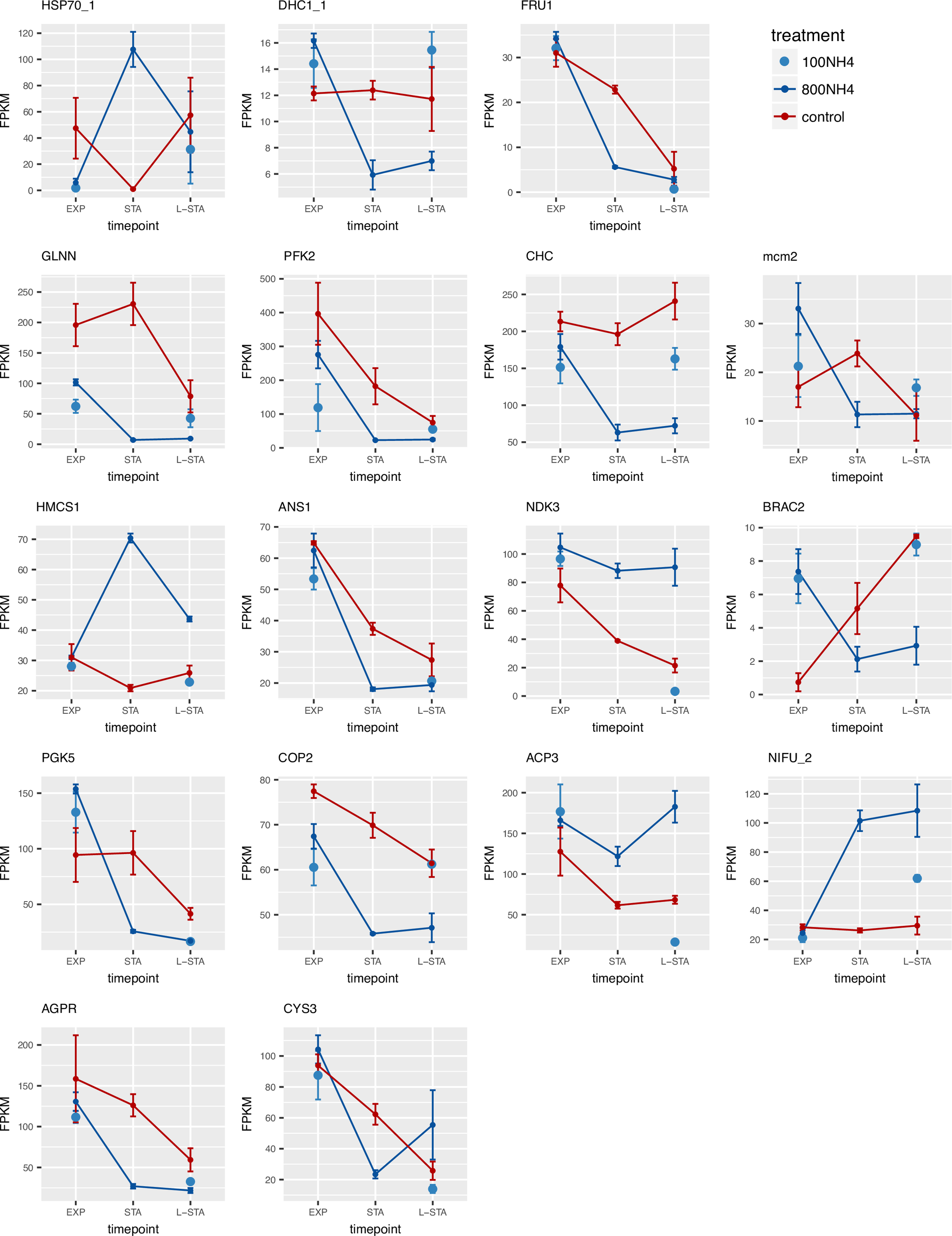

Supplement: S6 Fig — (TIF) [file pone.0181098.s006.tif]
